# Incidence of Histoplasmosis in a Cohort of People with HIV: From Estimations to Reality

**DOI:** 10.3390/microorganisms9122596

**Published:** 2021-12-16

**Authors:** Narda Medina, Juan Luis Rodriguez-Tudela, Luis Aguirre, Luis R. Salazar, Osmar Gamboa, Oscar Bonilla, Juan C. Pérez, Eduardo Arathoon, David W. Denning, Ana Alastruey-Izquierdo

**Affiliations:** 1 Asociación de Salud Integral, Guatemala City 01001, Guatemala; nardagab@gmail.com (N.M.); luaguirre9005@gmail.com (L.A.); luisro1992@gmail.com (L.R.S.); osmar.gamboa@asigt.org (O.G.); earathoon@hotmail.com (E.A.); 2 Global Action Fund for Fungal Infections, 01564 Geneva, Switzerland; jlrodrigueztudela@gaffi.org (J.L.R.-T.); ddenning@manchester.ac.uk (D.W.D.); 3 Clínica Familiar “Luis Ángel García”, Hospital General San Juan de Dios, Guatemala City 01001, Guatemala; oscar.bonilla@asigt.org (O.B.); jcpsmh@gmail.com (J.C.P.); 4 The National Aspergillosis Centre, University Hospital of South Manchester, Manchester M23 9LT, UK; 5 Manchester Academic Health Science Centre, School of Medicine, The University of Manchester, Manchester M23 9LT, UK; 6 Mycology Reference Laboratory, National Centre for Microbiology, Instituto de Salud Carlos III, 28222 Madrid, Spain

**Keywords:** histoplasmosis, antigen, HIV, opportunistic infections

## Abstract

Among people with HIV, histoplasmosis represents an important cause of mortality. Previous studies provided estimates of the disease incidence. Here, we compared those estimates with the results obtained from a screening program implemented in Guatemala, which included histoplasmosis detection for people with HIV. To compare the results of this program with previous estimations, a literature search was performed and reports concerning histoplasmosis incidence were analyzed. The screening program enrolled 6366 patients. The overall histoplasmosis incidence in the screening program was 7.4%, which was almost double that estimated in previous studies. From 2017 to 2019, the screening program showed an upward trend in histoplasmosis cases from 6.5% to 8.8%. Histoplasmosis overall mortality among those who were newly HIV diagnosed showed a decrease at 180 days from 32.8% in 2017 to 21.2% in 2019. The screening approach using rapid diagnostic assays detects histoplasmosis cases more quickly, allowing a specific treatment to be administered, which decreases the mortality of the disease. Therefore, the use of these new techniques, especially in endemic areas of histoplasmosis, must be implemented.

## 1. Introduction

In recent years, histoplasmosis has been recognized as an important opportunistic infection among people with HIV in Latin America [1,2]. However, until the development and commercialization of the antigen detection assay, diagnosis was cumbersome. It involved invasive procedures, which were unavailable in many low- and middle-income countries [3,4]. Classical diagnostic procedures, such as blood culture, were the gold standard for diagnosis of histoplasmosis. However, they require complex laboratory infrastructure (Biosafety Level 3), are time-consuming, and have a limited sensitivity to detect the disease, from 36% to 77% [1,5]. In this context, accurate diagnosis was challenging. Recently, the antigen detection assay has shown a high analytical sensitivity and specificity of 98% and 97%, respectively [1,4]. Therefore, results from this test provide a more accurate picture of the real disease burden.

In Guatemala, since 2017, a screening program for people with HIV has provided the detection of histoplasmosis, among other opportunistic infections. This program has allowed the real incidence of this disease to be quantified in people with HIV. Here, we compare histoplasmosis incidence published in the literature and the results of the screening program implemented in Guatemala from 2017 to 2019, showing the implications that the different approaches have in the care of the population with HIV.

## 2. Materials and Methods

### 2.1. Study Design and Participants

A literature search was performed in July 2021 using MEDLINE, Scielo, and Google Scholar to identify published studies in which the incidence of histoplasmosis in Guatemala was reported. Then, we compared the absolute differences in histoplasmosis incidence between these analyses and data obtained from the screening program performed from 2017–2019. A detailed description of the program was published previously [6,7]. Briefly, patients were enrolled at healthcare facilities, which are part of a national network that covers 81.2% (13 out of 16) of the sites that provide care to HIV-infected patients in the country. The program included three groups of HIV patients: (i) newly diagnosed; (ii) patients who have not been receiving antiretroviral treatment (ART) for >90 days but who returned to care (Return/Restart); and (iii) patients on ART with symptoms of opportunistic infections (ARV treatment). The clinical samples for the screening were sent from the healthcare facilities to a central diagnostic laboratory hub located in Guatemala City. Patients were screened for tuberculosis (TB), nontuberculous mycobacteria (NTM), histoplasmosis, and cryptococcal disease, independently of their CD4 cell count. A diagnosis of histoplasmosis was considered when the antigen detection, culture, or PCR test was positive. Advanced HIV disease (AHD) was defined as having a CD4 count of <200 cells/mm^3^. In this analysis, we describe histoplasmosis trends from 2017 to 2019 and the reported numbers of deaths at 180-day follow-up.

Demographic data were collected in the healthcare facilities using a standard electronic form. Patients received ART and treatment for opportunistic infections in accordance with the national guidelines [8]. Histoplasmosis refers to a positive result for Isolator blood culture, urine antigen, and/or a histoplasma PCR, not subacute disseminated or chronic pulmonary histoplasmosis, which were not estimated or documented.

### 2.2. Statistical Analysis 

Baseline characteristics were compared with the chi-square or Fisher’s exact test for categorical variable. The histoplasmosis incidence was assessed using the chi-square trend test. The statistical analysis was performed using SPSS (IBM Iberica, Madrid, Spain)and graphics were developed in Microsoft Excel. Incidences were calculated from the patients tested globally and for each group of patients using percentages. Differences between incidences obtained in the literature and this study were compared in absolute numbers. Histoplasmosis-related mortality was analyzed with Kaplan–Meier at 180-day follow-up. A *p* value of <0.05 was considered statistically significant. 

## 3. Results

**Figure 1:**
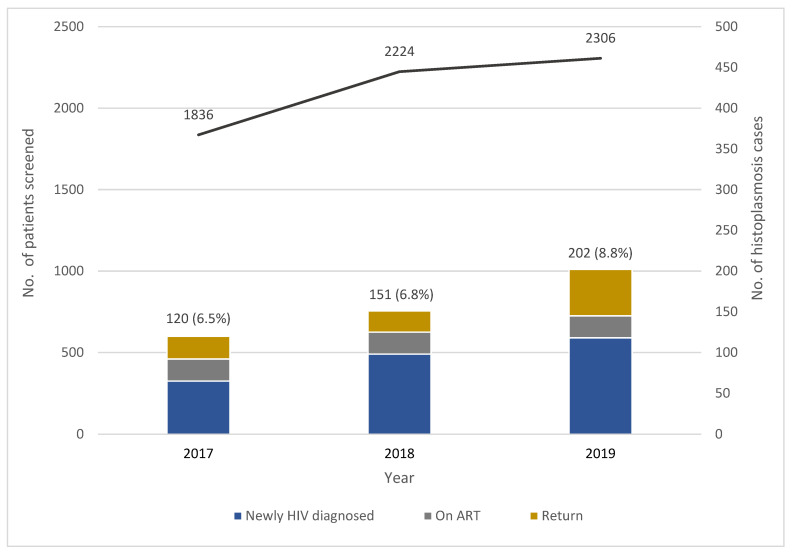
Annual screening patient numbers and histoplasmosis cases detected in the OI program.

Two previous publications reported histoplasmosis incidence among people with HIV in Guatemala. In 2018, Adenis et al. estimated an overall histoplasmosis incidence of 1.48% for the Latin American region, with Guatemala showing an incidence of 4.16%. This accounted for 2676 cases [9]. Previously, in 2015, Medina et al. had estimated the incidence in 3.8% [10]. However, case incidence of 7.4% in the screening program is about two-fold or an additional 1523 cases. A total of 6366 patients were included in the screening program. The overall incidence per year found was 6.5% in 2017, 6.8% in 2018, and 8.8% in 2019 showing a statistically significant upward trend in the absolute number of histoplasmosis cases (*p* = 0.005). However, there were differences among the three group of patients. Thus, newly diagnosed HIV patients had a histoplasmosis incidence of 8.5%, patients who returned to care of 8.3%, and those on ART of 4.8% (Figure 1). We observed a steady annual increase in histoplasmosis incidence within the newly diagnosed HIV patients and patients on ARV, while the other group, i.e., patients who returned to care, showed a decrease in 2018. Table 1 shows the annual incident histoplasmosis cases per type of patients. Considering histoplasmosis cases, the urine antigen was positive in 69% (311 out of 452), the PCR in 60% (249 out of 416), and the isolator blood in 35% (123 out of 353). One hundred and sixty-five cases were diagnosed by combining assays. 

At enrollment, CD4 cell counts were available for 4625 patients (72.5%); of those, 2235 (48.3%) had AHD. The incidence of histoplasmosis in AHD was 11.9% among the newly diagnosed HIV patients in comparison with 2.7% in those without AHD. In the group of patients on antiretroviral therapy and those who returned to care with AHD, the histoplasmosis incidence was 8.6% and 11.5%, respectively.

Among those who had histoplasmosis, most patients were male (65.8%) between 28 to 42 years old. Sexual orientation and area of residence were found to be associated with histoplasmosis status through the chi-squared analysis (*p* < 0.005). Incidence of histoplasmosis in rural areas was higher than in urban areas (9.4% vs. 5.4%, *p* < 0.0001) and among heterosexual men vs. men who have sex with men (8.1% vs. 4.9%, *p* < 0.0001). Coinfection was found in 74 (15.6%) out of 473 patients diagnosed with histoplasmosis. The most common dual or triple infection was histoplasmosis plus tuberculosis with 42 cases (56.8%), followed by histoplasmosis plus cryptococcosis with 22 cases (29.7%), histoplasmosis plus NTM with 7 cases (9.5%), and histoplasmosis–cryptococcosis–tuberculosis with 3 cases (4.1%). *Pneumocystis* pneumonia and esophageal candidosis data were not systematically collected during these years. Table 2 summarizes several characteristics of the screened patients. 

**Figure 2:**
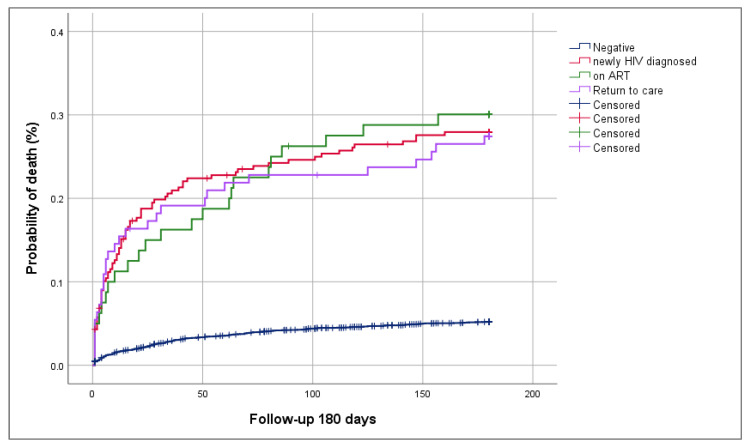
Probability of death in patients with histoplasmosis and HIV per type of patient.

Four hundred and sixty-nine patients with histoplasmosis were followed up at 180 days; of these, 131 deaths (27.9%) were reported. Eighty-seven (66.4%) of these deaths occurred in the first 30 days. Figure 2 shows the overall probability of death in patients with histoplasmosis per type of patient, and Table 3 display this mortality per year. Among newly diagnosed HIV patients, there was a decline in histoplasmosis deaths along with the overall decline in mortality (32.8% in 2017 to 21.6% in 2019; *p* = 0.0626). However, patients who returned to care and those on ARV showed a mortality increase at 180 days in 2018 and decline in 2019. The number of patients receiving histoplasmosis treatment in those newly diagnosed with HIV increased from 84.6% in 2017 to 92.4% in 2019; whereas in those who returned to care, this number increased slightly from 82.1% in 2017 to 86% in 2019, and in patients on ART, it increased from 74.1% in 2017 to 85.2% in 2019. Amphotericin B deoxycholate and itraconazole were the drugs available in the country during the study period. 

## 4. Discussion

Fungal diseases are generally neglected for many reasons. Diagnosis is often complicated, although new detection methods have become available. Furthermore, the content of educational programs concerning fungal diseases is a minor topic in medical schools and even in continuous educational postgraduate programs. This means that clinical suspicion of fungal disease is limited even though many of these infections are life threatening. This low level of suspicion implies that clinicians do not obtain an appropriate diagnosis and consequently many laboratories do not have the expertise and techniques required for prompt detection or exclusion of serious fungal disease. The project performed in Guatemala illustrates this situation. Using a screening strategy in high-risk patients with HIV infection, irrespective of the clinical suspicion and immunological status, we found that histoplasmosis was routinely underestimated. Our program provides much more accurate incidence data, allowing for the correct tools to diagnose and treat this disease to be allocated in the country. Guatemala has limited resources, but appropriate rapid screening for histoplasmosis is clearly worthwhile in HIV patients.

The use of a central diagnostic laboratory hub for the screening program, as opposed to local laboratories, should be analyzed carefully. A centralized laboratory, such as that implemented in Guatemala, offers advantages such as a lower cost per sample, more expertise, improved test quality control, and centralized epidemiological data. On the other hand, local laboratories are closer to patients, making results more easily available. However, it is important to consider the number of samples that local laboratories run per week and the availability of a quality control program to ensure the correct performance of the whole diagnostic process. Successful national or regional implementation through a central laboratory must provide a good system for delivering samples from the peripheral centers linked with a structured program to facilitate point-of-care tests (when they are available) at the bedside or in clinic. 

Over the study period, we observed a general upward trend in histoplasmosis detection. In 2012, the national epidemiological report only identified 32 cases of histoplasmosis in the country [10]. In the screening program, a diagnosis of histoplasmosis was reached in 158 people per year on average, from more than 1800 tests performed. Access to diagnosis is the key change in the care of people with HIV in Guatemala, as it provides more accurate epidemiological data and allows for better clinical care. A key component of the project was the training program. Unexpected diagnostic results from certain tests made clinicians more aware of histoplasmosis, and encouraged them to request more tests and treat patients quickly and appropriately, both for histoplasmosis alone and in cases with dual or triple infections. The increasing histoplasmosis incidence shown in this analysis should spur others to increase efforts to address all cases with rapid diagnostic testing.

As a result of comparing our findings with the previously published data, it can be seen that the introduction of new assays such as the antigen *Histoplasma* detection test should be mandatory. This assay is affordable, provides rapid results, and the clinical sample tested is urine, which is easily collected. The literature data show an underestimation of histoplasmosis cases that may be related to the use of tests with a low sensitivity, the nature of the studies, and the lack of clinical suspicion. Histoplasmosis incidence in the screening program was about two-fold higher than the previous estimate by Adenis et al. (7.4% vs. 4.16%) [9]. Guatemala is known as a hyperendemic country with one of the highest incidences of histoplasmosis in Latin America. Our study shows an excess of 1523 cases of histoplasmosis among people with HIV in Guatemala, as compared to prior estimates. Other countries such as Guyana (2.76%), Venezuela (2.90%), Argentina (1.89%), and the Central America region were also cataloged as having high incidence levels (≥1.5%) [9], but the annual incidence is probably underestimated also. Unavailability of laboratory tests and a lack of screening strategies in patients at high risk of opportunistic infections are related to the underestimation of the burden of diseases. Moreover, in people with HIV, histoplasmosis symptoms are nonspecific and are often indistinguishable from other diseases like tuberculosis. Examples of common clinical features that can be confused with other disease entities include nonspecific respiratory complains and pulmonary infiltrates (TB, *Pneumocystis*, or bacterial pneumonia), pancytopenia (HIV, visceral leishmaniasis, lymphoma, drug toxicity), hepatosplenomegaly (leishmaniasis, lymphoma, disseminated TB), diarrhea and weight loss (HIV, disseminated NTM infection, intestinal infection), and others. As our work shows, histoplasmosis can be present with other HIV complications in the same patient. The frequency of this co-occurrence has been reported as being from 9% to 38% [11,12,13]. 

We also found that the incidence of histoplasmosis is almost double in rural as compared to urban areas of Guatemala. Many studies are conducted in urban settings only. In 1960, a histoplasmin skin survey was performed showing an overall reactivity in the general population of 57.2% [14], with the rural areas exhibiting the highest frequencies, which is in accordance with our current findings. According to the national HIV continuum report, facilities included in the screening program provide healthcare attention to approximately 60% of population with HIV in the country [15]. Therefore, our epidemiology data represent a robust estimate and indicate that diagnostic services need to reach rural areas for histoplasmosis. 

The screening program provided access to diagnosis independently of the CD4 cell count. If a limit of <200 cells/mm^3^ had been used, 27.5% of the patients would not have been screened. In our study, 92% of the histoplasmosis cases occurred in patients with <350 CD4 cells. Our findings also showed that the overall histoplasmosis mortality was 29.6% in 2017, 33.3% in 2018, and 22.8% in 2019. Excluding the 2018 value, there was an overall reduction in mortality from 2017 to 2019 of 6.8%. The reduction in histoplasmosis mortality was observed in those with newly diagnosed HIV, from 32.8% in 2017 to 21.2% in 2019. Clearly, access to histoplasmosis diagnosis is essential to guide appropriate treatment decisions. Patients who returned to care and those on ART showed an unexpected increase in mortality for histoplasmosis in 2018. Although there is no clear explanation for this, the unavailability of rapid immunological and virological status results of patients already entered in ARV programs could have influenced the speed of diagnosis requests and treatment approaches. 

This study has certain limitations. First, differences between healthcare facilities as regards the way in which they actively search for patients to return to treatment and the way in which patients on ARV are managed might have influenced the screened group. Second, we did not collect patient clinical information; therefore, we were not able to determine the number of incident cases that presented with and without symptoms, and with which clinical presentation. Third, the histoplasmosis incidence calculated in this study was compared with estimates that were extrapolated to the HIV-positive population, and therefore it is not completely comparable; however, this is the only study available and we believe that due to the high number of patients screened, the numbers gathered are quite robust and give an idea of the real incidence. Despite these limitations the program gathered a significant amount of data from active screening, providing a new perspective for estimating the burden of disease and allowing access to diagnosis for life-threatening infections in a high-risk population of patients. Therefore, we strongly recommend a screening approach in high-burden locations and countries for several reasons: (i) the high rate of coinfections in this population; (ii) the difficulties in distinguishing tuberculosis and other conditions; and (iii) as an indirect tool for continuing the education of clinicians, which forces them to make a careful evaluation of the patient to confirm unexpected laboratory diagnoses and to take treatment decisions to save patients’ lives. 

## 5. Conclusions

There is a clear need to reinforce diagnostic laboratories in low- and middle-income countries. The new diagnostic tools with a high sensitivity and specificity have changed the landscape of fungal infection diagnosis and can be implemented very quickly. Training of clinicians is also required alongside the introduction of new tests. This can take a long time if the disease is unfamiliar. By introducing screening, we can quickly improve the quality of patient care as it requires a clinical response when tests are positive. Strategic introduction of diagnostic laboratory hubs with a screening approach should accelerate clinical care improvements. In addition, regional or national diagnostic laboratory hubs can establish the burden of several diseases and develop rational screening programs according to early results. The proper delivery of clinical samples to the hub and training and quality control programs could provide global access to diagnosis and decrease the mortality of fungal diseases everywhere, as the Guatemala program has shown. Nowadays, diagnostic technique variability is minimal compared with the variability of medical practice. Diagnosis of life-threatening diseases should not rely solely on clinical training but should be reinforced by the provision of valuable diagnostic information. The implementation of diagnostic laboratory hubs with screening programs would improve patient care more quickly and with a lower variability than medical training programs alone, for most fungal diseases. Laboratory diagnostic hubs are also a cost-effective means of providing high-quality access to the diagnosis of fungal diseases.

## Figures and Tables

**Table 1:** Incidence of histoplasmosis among different groups of patients, by year.

Type of Patient	Screened Patients	Overall Incidence	Year 2017	Year 2018	Year 2019
Newly diagnosed HIV	3322	8.5%	7.5%	8.3%	9.3%
On ART	1704	4.8%	4.1%	4.6%	5.9%
Return to care	1338	8.3%	9.2%	5.8%	9.8%
Overall	6366	7.4%	6.5%	6.8%	8.8%

ART = antiretroviral therapy.

**Table 2:** Baseline characteristics of patients screened for histoplasmosis.

Characteristics	Number of Patients Screened	Histoplasmosis (n = 473)		Non-Histoplasmosis (n = 5893)	
		n	%	n	%
Sex					
Male	4049	311	65.8%	3738	63.4%
Female	2252	158	33.4%	2094	35.5%
Transsexual	65	4	0.8%	61	1.0%
Age (years), median (IQR)	6360	35	(28–42)	34	(27–43)
Sexual orientation					
Heterosexual	4796	389	82.2%	4407	74.8%
Homosexual	1083	53	11.2%	1030	17.5%
Bisexual	371	19	4.0%	352	6.0%
Unknown	116	12	2.5%	104	1.8%
Residence					
Urban	3015	163	35.2%	2852	49.8%
Rural	3180	300	64.8%	2880	50.2%
Ethnic group					
Ladino	4909	368	77.8%	4541	77.1%
Mayan	914	67	14.2%	847	14.4%
Other	45	0	0%	45	0.8%
Unknown	498	38	8.0%	460	7.8%

**Table 3:** Histoplasma mortality at 180-day follow-up among HIV patients.

Type of Patient	Overall Mortality	Mortality by Year		
		2017	2018	2019
Newly diagnosed HIV	27.6	32.8	32.0	21.2
On ART	30.0	30.8	40.7	18.5
Return to care	27.3	21.4	30.8	28.6

## Data Availability

Not applicable.
